# Supplementary material for: Impact of HCV Eradication on Lipid Metabolism in HIV/HCV Coinfected Patients: Data from ICONA and HepaICONA Foundation Cohort Study
Source: Viruses. 2021 Jul 19;13(7):1402. doi: 10.3390/v13071402 (PMC8310285; doi:10.3390/v13071402)
Supplement: Supplementary file 1 [file viruses-13-01402-s001.zip › viruses-1275767-supplementary.pdf]

Figure S1. Panel A and B. Directed acyclic graphs depicting the underlying assumptions of the ANCOVA models in order to estimate the cause effect of exposure on outcome.

A

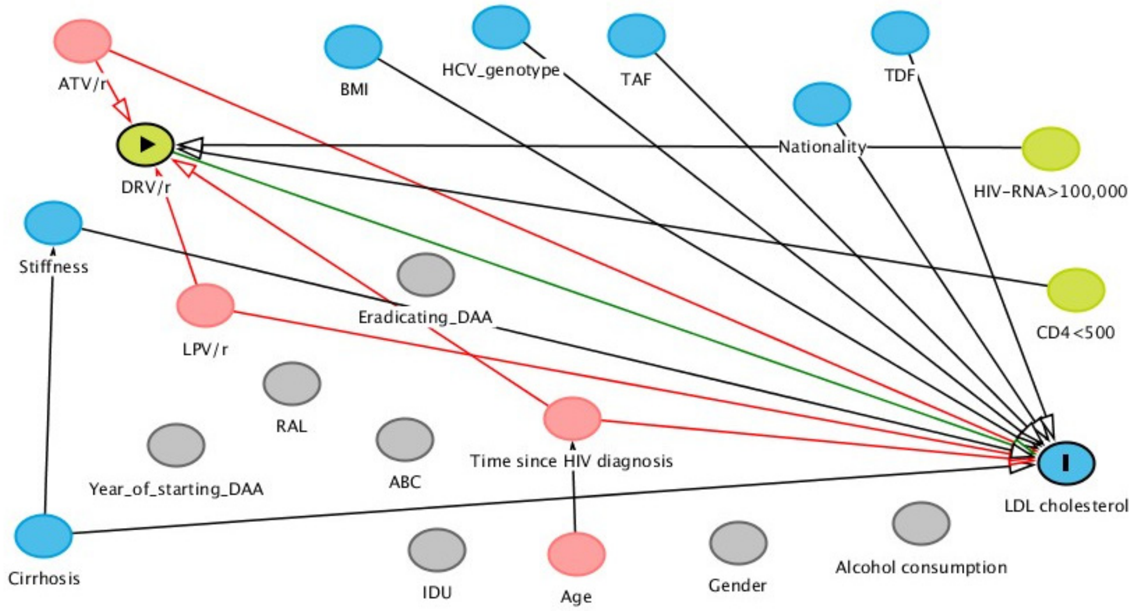

B

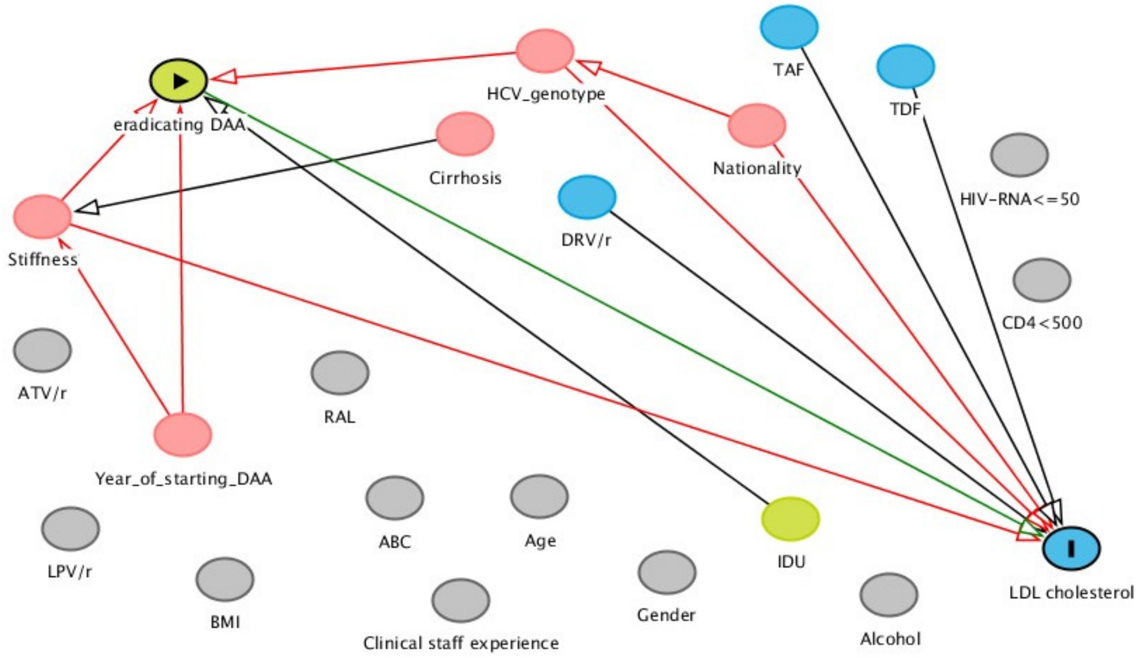

Table S1. Protocol codes and dates of approval of the study by Institutional Review Boards (or Ethics Committees) of all participants Centers.

Center Code	IRB name	Approval Date	Approval Protocol N.
A00	Comitato Etico Locale per la Sperimentazione Clinica dell'Azienda Ospedaliera Luigi Sacco - Via G.B. Grassi,74	20 December 2007	10/08/69/07 AP

	- 20157 Milano		
A01	Comitato di Bioteica della ASL Sassari - Via Monte Grappa, 82 - 07100 Sassari	10 December 2007	348/L
A04	Comitato di Bioteica dell'Azienda Ospedali Riuniti di Bergamo - Largo Barozzi,1 - 24128 Bergamo	15 October 2007	1932
A05	Comitato Etico dell'Azienda Osedaliero Universitaria di Cagliari - Via San Giorgio,12 - 09124 Cagliari	15 November 2007	261/07/CE
A06	Comitato Etico locale dell'Azienda USL 10 di Firenze - Ospedale Pietro Palagi - Viale Margherita,41 50125 Firenze	07 September 2007	0132246/67GP
A07	Comitato Etico Locale per la Sperimentazione Clinica dell'Azienda Ospedaliera Luigi Sacco - Via G.B. Grassi,74 - 20157 Milano	20 December 2007	10/08/66/07 AP
A15	Comitato Etico dell'Azienda Ospedaliera Universitaria "San Martino" di Genova - Largo Rossana Benzi,10 - 16132 Genova	09 November 2007	115/2007
A12	Comitato Etico Palermo 1 - AOUP Policlinico Giaccone - Via del Vespro, 129 - 90127 Palermo	18 January 2016	V2016
A13	Comitato Etico dell' Azienda Ospedaliera-Polo Universitario San Paolo - Via A. di Rudinì,8 - 20142 Milano	28 November 2007	44 CE/MA/CM
A14	Comitato Etico dell'Azienda Ospedaliera San Gerardo di Monza - Via Pergolesi,33 - 20052 Monza	25 October 2007	NA
A16	Comitato Etico Locale per la Sperimentazione Clinica dei Medicinali dell'Azienda Ospedaliera Universitaria Senese di Siena - Le Scotte - 53100 Siena	17 March 2014	NA
A17	Comitato Etico Indipendente Azienda Ospedaliero Universitaria di Cagliari - Via Ospedale 54 – 09124 Cagliari	30 January 2018	PG/2018/1445
A18	Comitato Etico Regionale per la Sperimentazione Clinica della Regione Toscana Sezione: AREA VASTA CENTRO	16 July 2019	1188_oss
A19	Comitato Etico Milano Area 2 - Via Francesco Sforza 28 - 20122 Milano	19 February 2019	169_2019
B00	Comitato Etico della Fondazione Centro San Raffaele del Monte Tabor - Istituto Scientifico Ospedale San Raffaele - via Olgettina, 60 - 20132 Milano	12 July 2007	NA
B02	Comitato Etico dell'Azienda Ospedaliera Osepdale Niguarda Ca' Granda - Piazza Ospedale Maggiore, 3 - 20162 Milano	14 September 2007	NA
B03	Comitato etico di Area Vasta Romagna ed IRST - Viale Ghirotti, 286 - 47023 Cesena	19 September 2007	63687
B04	Comitato Etico per la Sperimentazione Clinica della Provincia di Vicenza - Via Ridolfi, 37 - 36100 Vicenza	12 September 2007	98/07
B05	Comitato Etico dell'Ente Ospedaliero Osepdali Galliera di Genova - Via delle Mura Cappuccine, 14 - 16128 Genova	18 July 2007	RP/246/CE
B06	Comitato Etico per la Sperimentazione Clinica dei Medicinali della Provincia di Venezia - Via Don Federico Tosatto, 147 - 30174 Venezia	08 November 2007	29/CE
B07	Comitato Etico dell'Azienda USL di Latina - Via Canova,2 - 04100 Latina	04 October 2007	111/350
B08	Comitato Etico Indipendente dell'IRCCS CRO di Aviano - Via F. Gallini, 2 - 33081 Aviano (PN)	14 November 2007	857/D
B10	Comitato Etico Unico per la Provincia di Parma - Via Gramsci, 14 - 43100 Parma	15 September 2007	26547

B11	Comitato Etico Provinciale per la Sperimentazione Clinica - Viale Tre Martiri 45100 Rovigo	17 June 2009	217/CEP
B12	Comitato Etico dell'Azienda Ospedaliero Universitaria Santa Maria della Misericordia di Udine - Via Colugna, 50 - 33100 Udine	28 August 2012	51460
B13	Comitato Etico dell'Azienda Sanitaria dell'Alto Adige - Via L. Bohler 5 - 39100 Bolzano	18 October 2018	77-2018
C00	Comitato Etico dell'Azienda Ospedaliera Spedali Civili di Brescia - Piazza Spedali Civili, 1 - 25123 Brescia	04 December 2007	NA
C02	Comitato Etico dell'Azienda Ospedaliera "Istituti Ospitalieri" di Cremona - Viale Concordia,1 - 26100 Cremona	28 February 2008	5522/08-RN/RV
C03	Comitato di Bioetica della Fondazione IRCCS Policlinico "San Matteo" - Viale Golgi, 19 - 27100 Pavia	25 June 2007	NA
C04	Comitato Etico Interaziendale delle AA.SS.LL di Torino - Strada dell'Arrivore 25/A - 10154 Torino	11 December 2007	000814/007B/2007
C05	Comitato Etico Interaziendale delle AA.SS.LL di Torino - Strada dell'Arrivore 25/A - 10154 Torino	11 September 2007	000658/007B/2007
C07	Comitato Etico dell'Azienda Ospedaliera "Ospedale di Lecco" - Via dell'Eremo 9/11 - 23900 Lecco	06 February 2008	NA
C09	Comitato Etico dell'Azienda Ospedaliera Ospedale di Circolo di Busto Arsizio - Piazzale Solaro,3 - 21052 Busto Arsizio (VA)	30 November 2007	42847
D00	Comitato Etico Indipendente dell'Azienda Ospedaliera-Universitaria di Bologna, Policlinico S.Orsola-Malpighi - Via Massarenti, 9 - 40138 Bologna	13 November 2007	125/2007/0/OSS
D01	Comitato Etico Provinciale di Reggio Emilia - Viale Risorgimento 57 - 42100 Reggio nell' Emilia	03 September 2007	24781
D02	Comitato Eitico per Sperimentazione Clinica della Provincia di Padova - Via Giustiniani, 1 - 35128 Padova	09 June 2016	3811/AO/16
D04	Comitato Etico Provinciale di Modena - Via del Pozzo 71 - 41100 Modena	06 November 2007	3039/CE
D05	Comitato Etico Provinciale di Ferrara - Corso Giovecca, 203 - 44100 Ferrara	24 July 2007	NA
D08	Comitato Etico dell'Azienda Ospedaliero-Universitaria Ospedali Riuniti Umberto I- G.M. Lancisi - G. Salesi di Ancona – Via Conca 71 – 60126 Ancona	29 November 2007	2070211
D09	Comitato Etico dell'Azienda Ospedaliero-Universitaria Ospedali Riuniti Umberto I- G.M. Lancisi - G. Salesi di Ancona – Via Conca 71 – 60126 Ancona	29 November 2007	2070212
D11	Comitato Etico Interzonale della ASUR zona Territoriale 8 di Civitanova Marche e Zona Territoriale 9 di Macerata - Via Santa Lucia 2 - 62100 Macerata	07 February 2008	60/INT/CEI/11263
D12	Comitato Etico per la Sperimentazione Clinica dei Farmaci della AUSL di Pescara - Via Fonte Romana, 14 - 65100 Pescara	18 January 2010	006
E00	Comitato Etico dell'Università Cattolica del Sacro Cuore - Policlinico Universitario Agostino Gemelli di Roma - Largo A. Gemelli, 8 - 00168 Roma	13 September 2007	589/A1376/CE/2007
E08	Comitato Etico dell'IRCCS Istituto Nazionale per le Malattie Infettive "Lazzaro Spallanzani" di Roma - Via Portuense, 292 - 00149 Roma	01 August 2007	38/2007
E05	Comitato Etico per la Sperimentazione dei Medicinali dell'azienda Ospedaliera-Universitaria Careggi di Firenze - Viale Pieraccini, 28 - 50139 Firenze	12 July 2007	NA

E06	Comitato Etico delle Aziende Sanitarie dell'Umbria di Perugia - Via della Rivoluzione, 16 - 06070 Perugia	21 July 2008	1296/08
E07	Comitato Etico per le Attività Biomedichedell'Università degli Studi "Federico II" di Napoli - Via Pansini, 5 - 80135 Napoli	24 February 2016	35-2016
E10	Comitato Etico dell'azienda Policlinico Umberto I di Roma - Viale del Policlinico, 155 - 00161 Roma	27 September 2007	1272
E11	3 RomaComitato Etico Indipendente dell'Azienda Ospedaliera Universitaria Policlinico Tor Vergata di Roma - Viale Oxford, 81 - 00133 Roma	27 November 2007	NA
E13	Comitato Etico Centrale IRCCS Lazio-Sezione IRCCS IFO-Fondazione GB Bietti - Via Elio Chianesi, 53 - 00144 Roma	24 June 2014	CE/38/14
E15	Comitato Etico Lazio 1 - Circonvallazione Gianicolense, 87 - 00152 Roma	13 January 2016	263/CELAZIO1
E16	Comitato Etico dell'Università "Sapienza" - Viale del Policlinico 155 - 00161 Roma	23 April 2015	3617
E17	Comitato Etico delle Aziende Sanitarie della Regione dell'Umbria - Via della Rivoluzione, 16 - 06070 Ellera di Corciano (PG)	16 March 2016	7925/16
F00	Comitato Etico Indipendente Locale dell'Azienda Ospedaliera Ospedale Policlinico Consorziale di Bari - Piazza Giulio Cesare,11 - 70124 Bari	28 November 2007	NA
F01	Comitato Etico dell'Azienda Ospedaliera Domenico Cotugno di Napoli - Via G. Quagliariello, 54 - 80131 Napoli	12 September 2007	32/2007
F06	Comitato di Etica per la Ricerca Biomedica dell'Università degli Studi "G. D'annunzio" e della ASL di Chieti - Via dei Vestini, 31 - 66013 Chieti	30 October 2007	7713/R
F08	Comitato Etico Interprovinciale Area 1 dell' Azienda Ospedaliero Universitaria "Ospedali Riuniti" di Foggia - Viale Pinto 1 - 71100 Foggia	12 September 2019	79/CE/2019
F13	Comitato Etico Catania 2 - Piazza S. Maria di Gesù, 7 - 95123 Catania	24 June 2014	NA
F15	Comitato Etico Catania 2 - Piazza S. Maria di Gesù, 7 - 95123 Catania	12 December 2017	42/2017/CECT2
F16	Comitato Etico di Messina - Via Consolare Valeria,1 - 98125 Messina	16 May 2016	20/16

Table S2. Participants' biomarkers according to pair contributions, using the latest value in the alternative window [+6;+12] months for T1-T2. DAA direct antiviral agent; BMI body mass index; LDL low-density lipoprotein cholesterol; HDL high-density lipoprotein cholesterol; GGT Gamma-glutamyl transferase; INR international normalized ratio

	**Pairs**													
	**T0-T1 (both pre-DAA treatment)**							**T1-T2 (pre and post DAA treatment)***						
**Biomarker**	**N**	**Mean1**	**SD1**	**Mean2**	**SD2**	**Difference**	**p-value**	**N**	**Mean1**	**SD1**	**Mean2**	**SD2**	**Difference**	**p-value**
BMI, kg/m2 Median (IQR)	139	26.5	27.2	25.0	21.0	-1.4	0.33	158	24.8	14.3	28.1	29.4	3.3	0.21
Blood glucose, mg/dL	551	96.21	30.04	94.88	26.43	-1.32	0.23	705	96.32	27.17	96.35	33.69	0.03	0.97
Creatinine *mg/dl*	658	4.7	5.5	4.6	7.5	-0.1	0.66	834	5.4	7.0	5.0	5.7	-0.4	0.08
Total cholesterol, mg/dL Median (IQR)	432	162.6	43.1	161.5	42.2	-1.2	0.37	487	161.1	40.9	183.7	40.3	22.6	<.01

LDL cholesterol, mg/dL Median (IQR)	170	96.6	38.8	94.6	37.7	-2.0	0.27	191	91.7	33.9	112.2	31.9	20.6	<.01
HDL cholesterol, mg/dL Median (IQR)	249	44.1	14.9	44.1	14.4	0.0	0.98	287	45.7	16.9	46.4	14.6	0.7	0.33
Tryglicerides Median (IQR)	431	137.2	83.0	134.6	78.1	-2.6	0.45	488	134.7	78.0	133.5	75.3	-1.2	0.73
ALT, *mg/dL*	667	83.79	78.14	78.24	62.42	-5.56	0.02	847	79.92	63.25	26.06	19.96	-53.9	<.01
AST, *mg/dL*	614	66.49	49.84	64.08	42.65	-2.41	0.13	788	66.96	46.41	27.05	13.15	-39.9	<.01
GGT, *mg/dL*	417	126.6	169.7	109.3	112.2	-17.3	<.01	505	111.1	119.2	47.27	91.67	-63.8	<.01
Total bilirubin, *mg/dL*	641	0.68	0.92	0.66	1.04	-0.03	0.49	788	0.66	1.02	0.53	0.75	-0.13	<.01
Platelets*/mmc*	670	173.8	77.7	170.4	70.9	-3.4	0.02	865	334.1	5027	179.2	72.6	-155	0.36
INR	98	0.95	0.35	0.98	0.34	0.03	0.55	121	1.01	0.35	0.98	0.41	-0.03	0.42
CD4 count, *cells/mmc*	731	689.2	706.7	860.6	3807	171.4	0.22	893	828.6	3498	699.9	540.2	-129	0.27
CD8 count, *cells/mmc*	669	923.9	498.8	926.0	546.3	2.14	0.87	767	923.9	585.8	987.1	643.7	63.20	<.01
CD4/CD8 ratio	669	0.79	0.43	0.94	3.02	0.15	0.21	767	1.16	5.71	1.44	16.77	0.28	0.66
HIV RNA *log10 cp/mL*	658	0.92	1.08	0.78	0.90	-0.15	<.01	819	0.77	0.90	0.70	0.88	-0.07	0.05

*Using the latest value in the alternative window [+6; +12]

Table S3. Pair analyses for all biomarkers, performed removing the 82 patients for which only pre-DAA biomarkers measurations were available. BMI body mass index; HCC hepatocellular carcinoma; LDL low-density lipoprotein cholesterol; HDL high-density lipoprotein cholesterol; GGT Gamma-glutamyl transferase; INR international normalized ratio.

**Biomarker**	**Pairs**													
	**T0-T1 (both pre-DAA treatment)**							**T1-T2 (pre and post DAA treatment)**						
	**N**	**Mean1**	**SD1**	**Mean2**	**SD2**	**Δ**	**p-value**	**N**	**Mean1**	**SD1**	**Mean2**	**SD2**	**Δ**	**p-value**
BMI kg/m2	139	26.5	27.2	25.0	21.0	-1.4	0.33	237	26.1	23.3	28.4	32.0	2.3	0.19
Blood glucose, mg/dL	551	96.21	30.04	94.88	26.43	-1.32	0.23	868	95.46	26.68	95.31	26.97	-0.16	0.82
Creatinine *mg/dl*	658	0.7	0.5	0.8	4.0	0.1	0.39	1027	0.8	3.2	0.7	0.7	-0.1	0.39
Total cholesterol *mg/dl*	350	162.0	43.3	160.4	42.2	-1.6	0.29	617	162.0	40.7	183.5	41.0	21.4	<.01
LDL cholesterol *mg/dl*	126	96.3	39.5	92.2	35.6	-4.0	0.04	235	91.9	34.3	114.4	32.2	22.4	<.01
HDL cholesterol *mg/dl*	189	43.2	15.0	43.4	14.6	0.3	0.70	358	45.9	16.7	46.5	14.3	0.5	0.41
Triglycerides *mg/dl*	350	141.9	86.8	138.1	81.8	-3.8	0.36	615	132.8	75.7	134.9	81.2	2.1	0.52
ALT, *mg/dL*	587	84.55	79.86	77.81	62.09	-6.74	<.01	1002	79.31	63.92	25.68	19.21	-53.6	<.01
AST, *mg/dL*	539	67.44	51.44	63.90	42.06	-3.53	0.04	946	66.19	46.96	27.32	15.75	-38.9	<.01
GGT, *mg/dL*	364	127.0	169.5	107.5	105.8	-19.5	<.01	638	108.3	116.9	47.41	91.58	-60.9	<.01
Total bilirubin, *mg/dL*	563	0.68	0.94	0.67	1.08	-0.02	0.70	944	0.67	1.02	0.55	0.79	-0.12	<.01
Platelets*/mmc*	589	171.8	79.1	168.9	72.0	-2.9	0.06	1010	313.2	4652	178.3	71.8	-135	0.36
INR	85	0.96	0.35	0.97	0.36	0.00	0.95	184	0.96	0.38	1.00	0.37	0.04	0.23
CD4 count, *cells/mmc*	649	687.8	734.2	862.0	4015	174.2	0.26	1013	805.2	3268	737.3	754.8	-67.9	0.51

CD8 count, *cells/mmc*	595	925.0	492.4	924.8	529.1	-0.13	0.99	874	915.0	553.3	959.9	576.5	44.86	<.01
CD4/CD8 ratio	595	0.78	0.42	0.94	3.20	0.16	0.22	874	1.13	5.35	2.54	49.80	1.41	0.41
HIV RNA *log10 cp/mL*	587	0.93	1.08	0.79	0.91	-0.14	<.01	931	0.78	0.90	0.69	0.90	-0.09	<.01
